# Cooperative Change in the Internal Dynamics of Streptavidin Caused by Biotin Binding

**DOI:** 10.1021/acs.jpcb.3c00427

**Published:** 2023-03-29

**Authors:** Mona Sarter

**Affiliations:** ISIS Neutron and Muon Facility, STFC Rutherford Appleton Laboratory, Chilton, Didcot OX11 0QX, U.K.

## Abstract

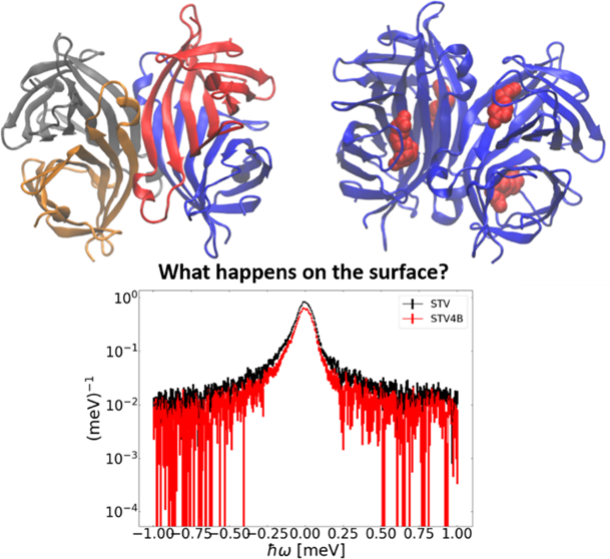

Protein–ligand interactions are of vital importance for biological functions. The biological function of proteins, such as ligand-binding, is strongly influenced by their dynamics. Quasielastic neutron scattering (QENS) was used to investigate the internal molecular dynamics of streptavidin (STV). QENS experiments to probe the internal dynamics were performed on a ps and 50–100 ps timescale using inverted geometry time-of-flight spectrometers. At the 50–100 ps timescale, the internal equilibrium motions of streptavidin proved to be unaffected by biotin (B) binding. However, on the ps timescale, suppression of jump-diffusion is observed even upon partial ligand saturation. This change indicates that the entire STV protein was affected by the population of one of the four binding sites, thus supporting a cooperative effect.

## Introduction

Protein–ligand interactions play an important role in biology.^1,2^ They control and provide signal transmission and physiological function and depend strongly on the protein’s dynamics.^3^ For a protein to display its full range of dynamics, the residues need to move freely, which is only the case for hydrated proteins.^4−6^ Therefore, to observe the full range of dynamics, especially larger amplitude motions and global diffusion, it is necessary to use solutions.^1,4,7^

Dynamic motions of proteins take place on different timescales 10^–14^ to 10^0^ s.^8,9^ Depending on the timescale selected for observation, different protein dynamics are visible.^9−13^ The different timescales correspond to different areas of the protein being involved in the dynamics.^9^ Therefore, the choice of timescale provides an opportunity to focus on the dynamics of different areas of a protein using quasielastic neutron scattering (QENS), even though the method itself does not provide explicit structural resolution.^14^ It does provide indirect information about local order though.^15^ Protein dynamics can be used as an additional source of structural information. Since different dynamics are localized on different areas of a protein, the general area involved in any given dynamic can be deduced.^16,17^

The current study addresses the influences that protein–ligand binding can have on protein dynamics. Streptavidin (STV) is a homotetramer that binds to the ligand B and forms a complex of four B molecules per STV homotetramer in the case of full saturation.^18^ It has a high binding affinity, as indicated by the dissociation constant *K*_d_ ≈ 4 × 10^–14^ M, making it one of the strongest noncovalent binding processes known in biology.^2,18−20^ Additionally, suppression of jump-diffusion dynamics shown by surface residues upon ligand saturation was previously observed.^21^ Here, the order of magnitude of the change in conformational entropy for STV was found to be the same as that of protein folding. This indicates that the whole protein instead of only the binding site is affected by the ligand-binding.^21,22^ The previously observed suppression of jump-diffusion in free STV upon saturation with B on the ps timescale supports this.^21^

In this paper, the question of how STV behaves for partial B saturation will be covered. This will address the open question of whether or not the STV + B binding is cooperative, since literature does not agree on that point.^18,23^ Good arguments have been presented for the cooperativity and non-cooperativity of the binding process, illustrating how difficult it is to get a detailed analysis of this extremely strong binding interaction.^18,23^ While this paper will make no claims about a change in *K*_d_ for each degree of biotin saturation, it will still discuss how the whole protein’s dynamic is affected by each additional binding pocket closing depending on the degree of saturation. This will also give more insight into how the previously discovered large change in Δ*S*_conf_ can coexist with the very small change in the crystal structure.^21,24,25^

## Methods

### Sample Preparation

As in previous work, STV (13–139) was bought from (ProSpec-Tany TechnoGene Ltd., Israel, Rehovot, catalogue number pro-791-c) at a molecular mass of 13 271.6 Da. The protein powder was dissolved in Mili-Q water and desalted using Pd-10 desalting columns (GE Healthcare, Chicago, IL), then it was lyophilized. This lyophilized powder was incubated in D_2_O (99.9 atom D) for 36 h to exchange labile protons with deuterium and then lyophilized again. Subsequently, it was lyophilized again and the resulting protein powder was dissolved in D_2_O-based buffer. The *H*/*D* exchange rate, as determined from the mass difference between the lyophilized hydrated and lyophilized deuterated protein powders, was 15.6%.^21^ B was purchased as a lyophilized powder with 99% purity (Sigma-Aldrich, 89555 Steinheim, Germany). Its concentration was determined by weight, using a molar mass of 244.3 g mol^–1^.

In total, five samples were used for the experiments. Ligand-free STV and STV with B ratios of 1 B per STV (STV1B), 2 B per STV (STV2B), 3 B per STV (STV3B), and 4 B per STV (STV4B), respectively. STV was reconstituted in the buffer at the required concentration. The buffer consisted of 25 mM Tris–HCl, 120 mM NaCl, 5 mM KCl, 3 mM MgCl, 99.9% atom D, pH 7.4, and STV was dissolved to a concentration of 60 mg mL^–1^, as determined by ultraviolet–visible (UV–Vis) (GeneQuant 1300) using the molar absorption coefficient  calculated from the STV sequence using ProtParam.^26^ Five separate aliquots were created from the solution. No further action was required for the STV sample. For the other samples, B was added in the required amount per sample to create STV1B, STV2B, STV3B, and STV4B. The strong binding affinity of STV and B was useful here since B was only added to the samples in the amount required to achieve the analyzed saturation. It was bound completely, and therefore, no free ligand existed in the solution, allowing for the buffer to only be measured once since no free ligand B ever existed.^27^

Neutron scattering experiments were performed using inverted geometry time-of-flight spectrometers IRIS^28^ and OSIRIS^29^ at the ISIS neutron and muon source. In order to determine the timescales, which correspond to each instrument’s energy resolution, the following relation was used . On IRIS, the energy resolution was 17.5 μeV full width at half-maximum (FWHM), leading to a time resolution of Δ*t* ≈ 40 ps allowing dynamics on the ∼50 to 100 ps timescale to be analyzed.^30^ Since the resolution gives the smallest unit of time that can be observed, it is possible to infer that dynamics on the ≈100 ps timescale are observed on IRIS.^31,32^ IRIS data were analyzed between *q* = 0.53–1.24 Å^–1^. On OSIRIS, the energy resolution was 99 μeV FWHM, which leads to a time resolution of Δ*t* ≈ 7 ps, allowing dynamics on the ∼1 to 10 ps timescale to be analyzed.^33,34^ OSIRIS data were analyzed between *q* = 0.60–1.44 Å^–1^. The samples were prepared in 1 mm annular Al sample cans, and the experiments were carried out at 298 K for both instruments. In addition to the samples, the buffer was measured, as well as a vanadium standard, which was used to obtain the instrument resolution.

### Data Analysis

All data were reduced and analyzed using Mantid software.^35^ The ConvFit interface of Mantid as well as self-written Python scripts executed in Mantid were used to perform the data analysis,^35,36^ and the buffer spectra were subtracted from the sample spectra.^15,37^ Because the samples were measured in a D_2_O-based buffer, the hydration layer was not explicitly subtracted, as it is not expected to provide a relevant contribution to the scattering function.^38^ The subtraction was performed based on eq 1.^15,37^
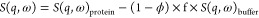
1with ϕ = *cV* as the volume fraction of the protein with concentration *c* and partial specific volume *V* = 0.71 mL g^–1^ and  as the factor correcting for neutron beam attenuation on IRIS and OSIRIS.

**Figure 1:**
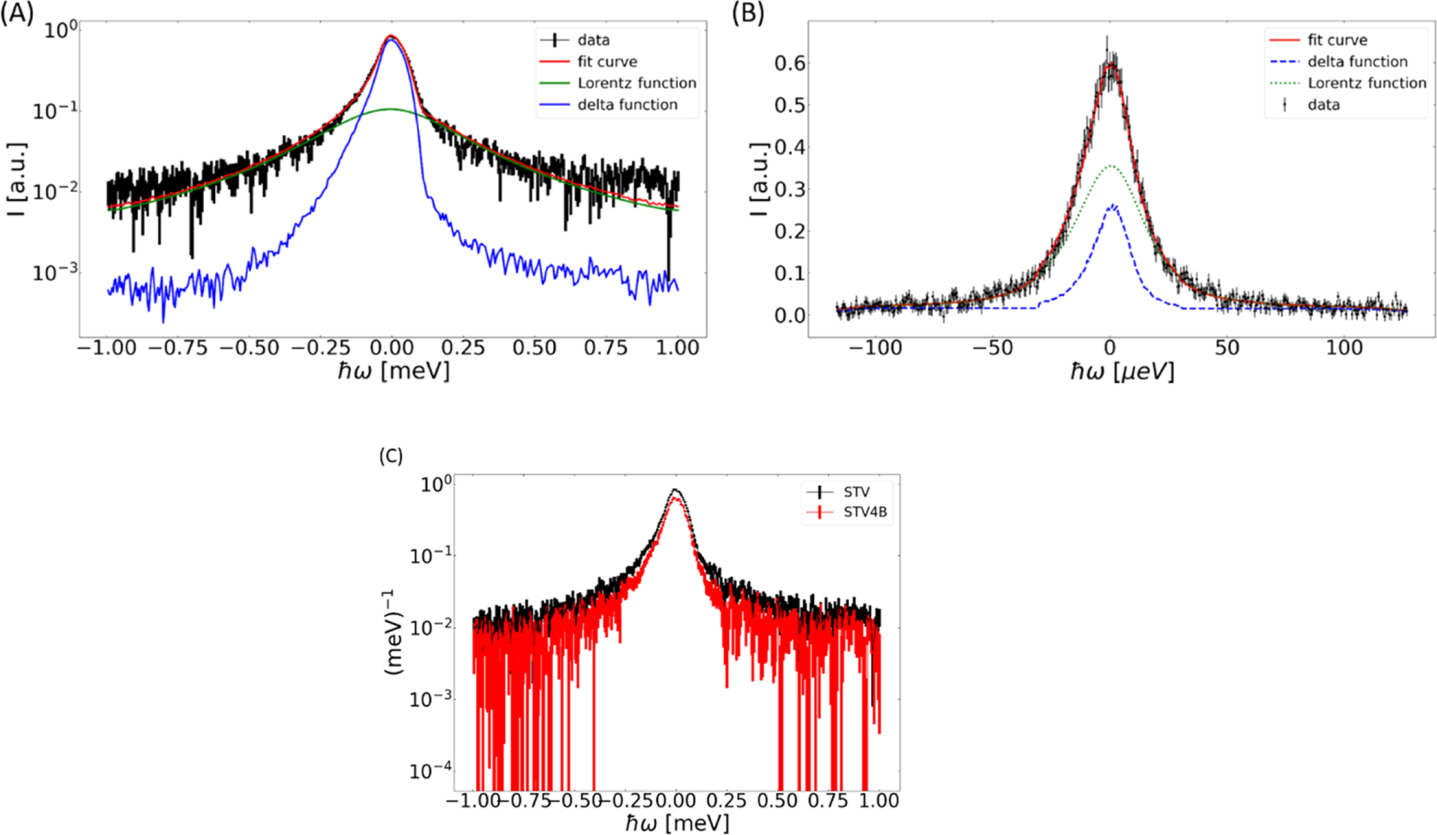
Examples of fits performed for the QENS spectra for STV. All data were collected at 298 K, while the fitted curves were numerically convoluted with the instrument resolution before plotting. Panel (A) shows data collected on OSIRIS at *q* = 1.23 Å^–1^ with the intensity displayed on a logarithmic scale for STV. Panel (B) shows an example of a fit performed for data collected on IRIS at *q* = 1.18 Å^–1^. Panel (C) shows an overlap of the QENS spectra for free STV and STV4B on OSIRIS at 298 K.

A phenomenological model was used to analyze the QENS scattering function. This model was numerically convoluted with the instrument resolution before being fitted to the scattering data.^39^ Data were analyzed with the scattering function *S*_I_(*q*, ω) eq 2, which exclusively reflects the internal dynamics of the protein. This was an appropriate choice because the observed timescale was too fast to resolve the slower center-of-mass diffusion of STV in solution.^21^
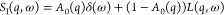
2*A*_0_(*q*) is the elastic incoherent structure factor (EISF), and Lorentzian *L*(*q*, ω) describes the quasielastic broadening of the spectrum.^8,15^
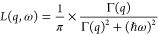
3Here, Γ(*q*) is the half-width half-maximum (HWHM) of the Lorentzian, and all spectra were fitted using eq 2, as displayed in Figure 1.

## Results

Neutron backscattering experiments were performed for ligand-free STV and at different ligand saturations (STV1B, STV2B, STV3B, and STV4B). QENS experiments were performed using the IRIS and OSIRIS spectrometers at the ISIS neutron and muon source.^31−34^

As discussed in the Methods section, global protein diffusion in solution was not measured using QENS because the timescales chosen for observation did not allow the center-of-mass diffusion of ≈6 A^2^ ns^–1^ to be fully resolved. The global diffusion was measured and calculated with HYDROPRO in previous experiments.^21^^100^In addition, the absence of aggregations at the analyzed STV concentrations was shown using DLS and SAS experiments in previous works.^21,40^ Instead, any potential influence of global diffusion on the spectrum was accounted for by a δ-function. This and the Lorentzian function were used to describe the scattering data, see eq 2.^41^

**Figure 2:**
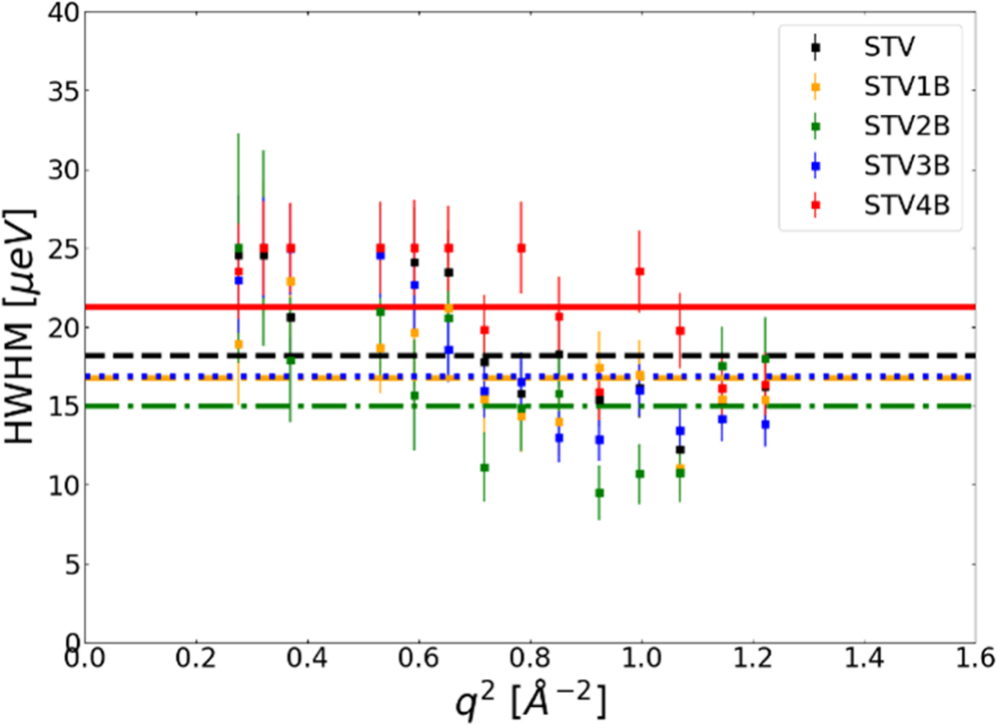
Line width Γ(*q*) (HWHM) as extracted from the QENS spectra plotted against *q*^2^ with IRIS in the pg002 setting for the 100 ps timescale. This shows the internal dynamics of STV at different degrees of saturation up to full saturation. All lines plotted are the uncertainty weighted average of the line width. The uncertainty is obtained for the fit parameters as the square root of the diagonal of the covariance matrix obtained for the fit parameters.

The internal dynamics of STV in solution were investigated by extracting the quasielastic peak broadening from the spectra and analyzing if Γ(*q*) is *q*-dependent. For data collected on IRIS, the results correspond to dynamics taking place on the 100 ps timescale.^42^Figure 2 shows that the quasielastic peak broadening Γ(*q*) is independent of *q*^2^ on the 100 ps timescale. In addition, the Γ(*q*) seems to fluctuate around a constant value.

The average values ⟨Γ⟩ in Table 1 were calculated as the weighted arithmetic mean since the Γ(*q*) values do not show any q-dependence. Different jump-diffusion models were tried and did not describe the data apart from the free STV on OSIRIS. This indicates a locally confined and restricted dynamic process. The error given on ⟨Γ⟩ is the standard deviation.
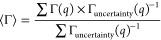
4From the weighted arithmetic mean ⟨Γ⟩, the correlation time τ_C_ is calculated using , the results are shown in Table 1.

**Table 1:** Internal Diffusion Coefficient, Correlation Times, and Residence Times Observed on the ps and 100 ps Timescale

	STV	STV1B	STV2B	STV3B	STV4B
*D*_int_ OSIRIS	2773 ± 300 Å^2^ ns^–1^				
τ_R_ OSIRIS	7.3 ± 0.2 ps				
τ_C_ OSIRIS		7.0 ± 1.8 ps	4.2 ± 2.2 ps	3.3 ± 0.9 ps	6.9 ± 1.6 ps
⟨Γ⟩ OSIRIS		0.09 ± 0.02 meV	0.16 ± 0.08 meV	0.20 ± 0.06 meV	0.10 ± 0.02 meV
τ_C_ IRIS	36.2 ± 9.0 ps	39.3 ± 9.1 ps	43.9 ± 19.9 ps	39.0 ± 11.6 ps	30.9 ± 5.4 ps
⟨Γ⟩ IRIS	18.2 ± 4.5 μeV	16.8 ± 3.9 μeV	15.0 ± 6.6 μeV	16.9 ± 5.0 μeV	21.3 ± 3.7 μeV

**Figure 3:**
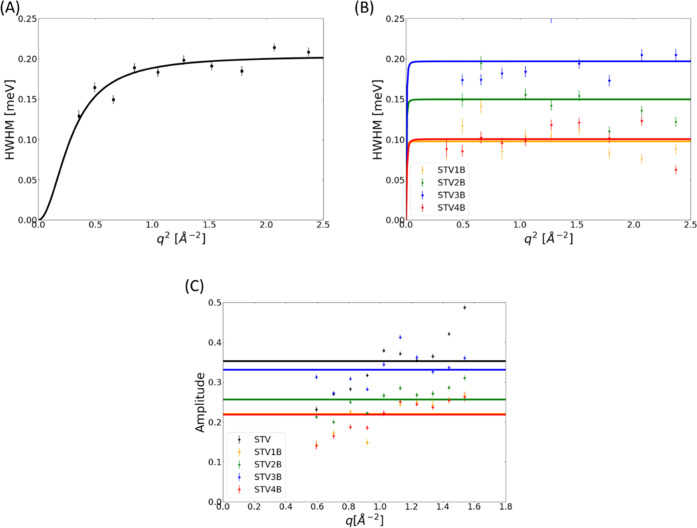
Line widths Γ(*q*) plotted against *q*^2^ for STV and different saturation states leading to the fully saturated STV4B state for data measured on OSIRIS in the pg004 setting on the ps timescale. In panel (A), the peak broadening vs q^2^ is plotted for the free STV; here, a jump-diffusive behavior of the peak broadening can be observed. In panel (B), peak broadening is observed for free STV, as well as for the complexes formed with *B* at different degrees of saturation. It can be observed that for STV1B-STV4B, the peak broadening fluctuates around a constant value instead of displaying jump-diffusive behavior. Panel (C) shows the amplitudes, with the uncertainties of the fit parameters as the error, of the Lorenzian fitted to the OSIRIS data. It can be observed that the weighted mean (calculated in accordance with the calculations for ⟨Γ⟩) is higher for the free STV and then follows the behavior of ⟨Γ⟩ for STV1B-STV4B.

Internal protein dynamics on the ps timescale were investigated using OSIRIS. Previous research found a jump-diffusive motion, eq 5, for STV, whereas the STV4B complex showed a locally constricted motion.^21^
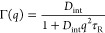
5On OSIRIS, both the free STV and the fully saturated STV4B complex were measured. In addition, the partially saturated intermediate states of STV1B, STV2B, and STV3B were measured. As shown in Figure 3A, free STV displayed the expected jump-diffusive behavior. Figure 3B also shows that in addition to the fully saturated STV4B, Γ(*q*) is *q*-independent starting from the first partially saturated state STV1B.

The jump-diffusive behavior of STV is best described using eq 3 for the *q*-dependence of the peak broadening with *D*_int_ as the internal diffusion coefficient and τ_R_ the residence time.^15,43^ For the ligand-bound state, a correlation time τ_C_ was determined using the average value ⟨Γ⟩ of the measured quasielastic broadening Γ(*q*), according to , the results can be seen in Table 1.^44^ While Figure 3B shows that the fit goes to 0 at *q* = 0 Å^–1^, this is only due to how the jump-diffusion is mathematically described with eq 5. For STV, however, the data are well described by the jump-diffusive model, as shown in Figure 3A.

**Figure 4:**
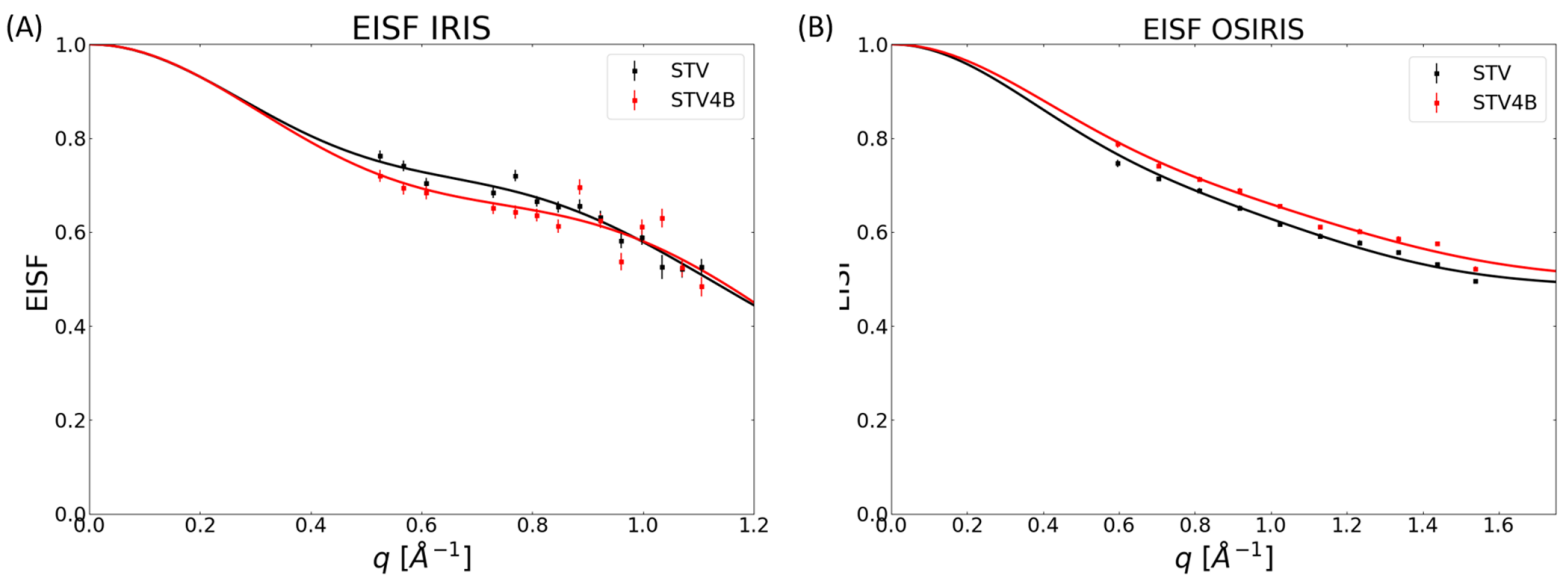
EISF (*A*_0_) vs *q* as measured on IRIS (A) and OSIRIS (B). While the difference in MSD between STV and STV4B matches previous results,^21^ the behavior of the intermediate states requires additional experiments and analysis using MD simulations to be fully understood.

In addition to the internal dynamics observed from the peak broadening, the EISF was used to calculate the mean square displacement (MSD) using the following equation for the modified Gaussian approximation.^45^
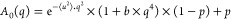
6Here, ⟨*u*^2^⟩ is the MSD, *p* the apparently immobile residues due to slower dynamics that are not resolved, and *b* accounts for the influence of multiple scattering. Analysis showed that the difference in MSD between STV and the STV4B complex is the same as that found in previous experiments, see Table 2 and Figure 4.^21^ In order to understand this behavior better and to analyze the intermediate states, additional investigation using MD simulations will be required, as well as longer counting times for data acquisition, which was outside the scope of this investigation. The results are shown here for STV and STV4B, respectively, and it can be seen by the difference in error that even the data for STV on OSIRIS proved difficult to fit with the required accuracy.

**Table 2:** MSD and *p* Factors Obtained from the EISF Fits^a^

	STV	STV4B
MSD IRIS [Å^2^]	2.54 ± 0.16	1.91 ± 0.12
MSD OSIRIS [Å^2^]	2.21 ± 0.27	1.88 ± 0.02

a MSD is related to the flexibility of proteins, while *p* describes the seemingly immobile section of the protein, meaning its dynamics are too slow to be observed.

## Discussion

The crystal structures of STV and the saturated complex STV4B show only minor differences.^24,25^ In addition, previously performed small-angle X-ray scattering (SAXS) and small-angle neutron scattering (SANS) experiments on free STV and the complex STV4B have shown that the protein in solution closely matches the structure and conformation of the crystal.^21,40^ By contrast, a pronounced change in the dynamics of STV upon partial or saturated binding of B is presented in this paper. Although the consistent structure between STV and STV4B indicates that no significant structural changes occur upon ligand-binding, the observed changes in dynamics, as well as the previously observed changes in conformational entropy, show that the dynamics of these residues are drastically altered by ligand-binding.^21^

Regarding the previously observed suppression of jump-diffusion for free STV upon ligand saturation on the ps timescale,^21^ it has now been shown that the jump-diffusive behavior is solely present on the ps timescale, while a *q*-independent behavior was observed for the peak broadening on the 100 ps timescale. The results from IRIS on the ∼100 ps timescale, see Table 1, show that for STV-STV4B, the results are identical within the error. This indicates localized or restricted motions, such as internal rotations of amino acid side groups.^1,12,46^ On the 100 ps timescales, these localized motions are most likely caused by the rotation of side chains and amino acids at the surface.^8,16,47−49^ Although τ_C_ for STV4B does seem to deviate to a slightly lower value, it is still within the error of the results for STV3B. If the correlation time τ_*C*_ is reduced for the fully saturated STV4B, this could be the result of the previously observed increased rigidity of the fully saturated STV4B.

Experiments on IRIS and OSIRIS provide information on internal dynamics that are two orders of magnitude different. With this difference in timescales, there are some areas for which dynamics might be observed on the ps and 100 ps timescale, while others will only occur on one of them. For free STV, a significant difference in behavior was observed between the two timescales. Therefore, all areas which are only mobile at one of the two timescales are likely to be the cause of this change in behavior. On the ps timescale, elastic vibrations of a protein’s globular region, as well as surface exposed residue rotation and motions, can be observed.^8,9^ While on the 100 ps timescale, in addition to these motions, torsional librations of buried groups, dynamics deeper in the protein, and the beginning of hinge-bending motions might be observed.^8,9^ The latter is highly improbable for STV, as the protein has a compact and stable tetrameric structure. Thus, a hinge-bending motion is not possible, considering that the structure of STV in solution matches the solid crystal structure.^40^ This leads to the conclusion that the jump-diffusive motion most likely takes place for the surface residues, as on the 100 ps timescale, no jump-diffusion is observed, thus ruling out areas closer to STV’s hydrophobic core, or its backbone from being involved in the jump-diffusion. All results are presented for only one temperature due to the limited availability of neutron beam time. The high thermal stability of STV, which has been previously reported, suggests that no significant structural conformational change occurs over a wide range of temperatures.^50,51^

A comparison of the results for the free, partially saturated, and fully saturated states on OSIRIS provides information on how B binding affects the dynamics of STV over the entire protein. First, insights into the change in protein dynamics upon ligand saturation were gained by examining the effect of partial saturation on the jump-diffusive behavior. For STV1B, it can be assumed that one B binds per STV tetramer.^22,50−101^ No jump-diffusive behavior was observed for STV1B; instead, the peak broadening Γ(*q*) showed *q*-independent behavior and fluctuated around one line. This indicates a localized restricted motion. In addition, this indicates that while B goes to one binding pocket for STV1B, it affects the dynamics of the whole tetramer. From this, a cooperative binding behavior can be inferred, as the whole of the protein is affected by the ligand instead of only the binding pocket, for which a structural change has been recorded. *Q*-independent behavior was also observed for the other partially saturated states STV2B and STV3B, as well as for the fully saturated STV4B complex. This change upon B binding to one of the four STV-binding pockets matches previous experimental results.^18,22^ Thermophoresis experiments found that upon STV1B formation, the hydrophobicity of STV1B was reduced compared to that of free STV.^22,53^ Hydrogen bonding sites in the free STV-binding pocket, as well as an increase in protein stiffness upon fully saturated binding, have been proposed as potential causes and as a sign of cooperativity. A change in thermal denaturation was also found at one B per STV-binding rate by González et al.^50,51^ Taking the change in fast dynamics of the surface residues upon 1B binding to STV into account, it now becomes apparent that this is caused by only one binding site being occupied. Therefore, the conclusion must be that some sort of whole-protein cooperativity affects the dynamics.

Since no changes in the dynamic behavior were found on the 100 ps timescale, it can be concluded that torsional librations and dynamics of groups lying deeper in the protein are not altered by the binding of B. This indicates that the surface residues and elastic vibrations of the globular region were restricted in their dynamics by ligand binding. Additional investigations are required to isolate the process that leads to the suppression of dynamics upon closing one binding pocket.

## Conclusions

While this paper can make no claim as to how or if the binding affinity of STV changes for different degrees of saturation, it has been successfully proven that the binding of one biotin affects the whole protein, well outside the binding site, and is therefore cooperative. Additional studies and molecular dynamic simulations will be required to understand all of the processes involved in the dynamics of this binding process. This study found conclusively that the binding of one biotin molecule to streptavidin (STV1B) suppresses the jump-diffusive motion on the ps timescale, which is indicative of cooperative binding, as the closing of one binding pocket affects the dynamics of surface residues over the whole protein.
